# Power output at the moderate-to-heavy intensity transition decreases in a non-linear fashion during prolonged exercise

**DOI:** 10.1007/s00421-024-05440-3

**Published:** 2024-03-14

**Authors:** Gabriele Gallo, Emanuela Luisa Faelli, Piero Ruggeri, Luca Filipas, Roberto Codella, Daniel J. Plews, Ed Maunder

**Affiliations:** 1 Sports Performance Research Institute New Zealand, Auckland University of Technology, Auckland, New Zealand; 2 Department of Neuroscience, RehabilitationGenoa, Ophthalmology, Genetics, Maternal and Child Health, University of Genoa, Viale Benedetto XV, 16100 Genoa, Italy; 3 Department of Experimental Medicine, University of Genoa, Genoa, Italy; 4 Department of Biomedical Sciences for Health, Università Degli Studi Di Milano, Milan, Italy; 5 Department of Endocrinology, Nutrition and Metabolic Diseases, IRCCS MultiMedica, Milan, Italy

**Keywords:** Durability, Cycling, Metabolism, Thresholds

## Abstract

**Purpose:**

The aims of this study were to: (i) describe the time course of the decrease in power output at the moderate-to-heavy intensity transition during prolonged exercise; (ii) investigate the association between durability of the moderate-to-heavy intensity transition and exercise capacity; and (iii) explore physiological correlates of durability of the moderate-to-heavy intensity transition.

**Methods:**

Twelve trained cyclists (age: 40 ± 8 y, $\dot{\text{V}}$O_2_peak: 52.3 ± 5.2 mL·min^−1^·kg^−1^) performed an exhaustive cycling protocol involving alternating incremental exercise tests to determine power output at the moderate-to-heavy intensity transition via the first ventilatory threshold (VT_1_), and 30-min bouts at 90% of the power output at the previously estimated VT_1_ in the rested state. The individual time course of VT_1_ was modelled using linear and second-order polynomial functions, and time to a 5% decrease in VT_1_ (Δ5%VT_1_) was estimated using the best-fitting model.

**Results:**

Power output at VT_1_ decreased according to a second-order polynomial function in 11 of 12 participants. Time-to-task failure (234 ± 66 min) was correlated with Δ5%VT_1_ (139 ± 78 min, *r*_s_ = 0.676, *p* = 0.016), and these were strongly correlated with absolute and relative rates of fat oxidation at specific exercise intensities measured during the incremental test performed in the rested state.

**Conclusions:**

These data: (i) identify a non-linear time course of decreases in the moderate-to-heavy intensity transition during prolonged exercise; (ii) support the importance of durability of the moderate-to-heavy intensity transition in prolonged exercise capacity; and (iii) suggest durability of the moderate-to-heavy intensity transition is related to fat oxidation rates.

## Introduction

Exercise physiologists have typically profiled endurance performance capabilities using maximum oxygen uptake ($\dot{\text{V}}$O_2_max), power output at the transitions between the moderate, heavy, and severe intensity domains, and movement economy or efficiency (Joyner and Coyle 2008; Poole et al. 2016). These variables are usually measured in a rested state without prior exercise, and then applied to performance profiling, training intensity and training load monitoring. However, these physiological parameters are not stable, but degrade over time during prolonged exercise (Passfield and Doust 2006; Noordhof et al. 2021; Stevenson et al. 2022; Clark et al. 2018). The time of onset and magnitude of these shifts with prolonged exercise has been termed ‘durability’ (Maunder et al. 2021).

Durability has implications for the application of physiological profiling data to prolonged training and competition. For example, it was recently shown that power output at the moderate-to-heavy intensity transition determined by the first ventilatory threshold and first lactate threshold decreased after 2 h of moderate-intensity cycling, with substantial inter-individual variation in the magnitude of the decrease (Stevenson et al. 2022). Therefore, performing prolonged exercise at an initially moderate-intensity power output may result in drift into the heavy-intensity domain. This has important implications as exercise performed in the moderate and heavy intensity domains elicits distinct metabolic, (Burnley and Jones 2018) autonomic, (Seiler et al. 2007) and adaptive (Stöggl and Sperlich 2015) responses. However, the time course of the decrease in power output at the moderate-to-heavy intensity transition during prolonged exercise has not been studied. These data may provide insights into how training programming and load monitoring may need to consider exercise duration. In addition, the importance of the individual time course of this response, and, therefore, durability, for prolonged exercise performance or capacity outcomes has not been assessed. These data would demonstrate the relevance of durability in endurance performance settings.

The physiological mechanisms that explain inter-individual variability in durability are not well-characterised. Carbohydrate ingestion during exercise negated the reduction in heavy-to-severe intensity transition power output after prolonged exercise, suggesting durability may be related to carbohydrate availability (Clark et al. 2019). Furthermore, cyclists exhibiting large increases in fatty acid oxidation during prolonged exercise, which likely reflects greater glycogen depletion due to the autoregulatory nature of carbohydrate metabolism (Hargreaves et al. 1995), saw the largest reductions in gross cycling efficiency (Stevenson et al. 2022). As muscle glycogen depletion appears to reduce the number of excitable fibres (Cairns and Renaud 2023), it is plausible that, during submaximal exercise, athletes capable of oxidising fat at higher rates preserve glycogen and muscle excitability, and are, therefore, more durable. A previous study reported no relationship between durability of the moderate-to-heavy intensity transition and the peak fat oxidation rate (PFO) during incremental exercise (Stevenson et al. 2022). Although a strong relationship between fasted, incremental exercise PFO and fat oxidation rates during prolonged, fed-state cycling at 80% of the first ventilatory threshold (VT_1_) has been reported (Maunder et al. 2022), PFO is not a direct measure of absolute or relative fat oxidation rates during all exercise durations and intensities. Therefore, it may be more appropriate to explore this hypothesis by assessment of relationships between durability and substrate oxidation rates in the initial periods of prolonged exercise at the specific exercise intensity.

Therefore, the aims of this study were to: (i) evaluate the time course of the decrease in power output at the moderate-to-heavy intensity transition during prolonged exercise, (ii) assess the relationship between durability of the moderate-to-heavy intensity transition and exercise capacity, and (iii) explore physiological correlates of durability of the moderate-to-heavy intensity transition. We hypothesised that: (i) power output at the moderate-to-heavy intensity transition would decrease in a non-linear fashion during prolonged exercise, and that considerable inter-individual variability in the time of onset of decrease would be present, (ii) durability of the moderate-to-heavy intensity transition would be correlated with exercise capacity, and (iii) durability of the moderate-to-heavy intensity transition would be correlated with absolute and relative rates of fat oxidation in the initial periods of prolonged exercise.

## Methods

### Participants

Twelve trained (McKay et al. 2022) cyclists (10 male, 2 female) participated in the present investigation (age: 40 ± 8 y, peak rate of oxygen uptake [$\dot{\text{V}}$O_2_peak]: 52.3 ± 5.2 mL·min^−1^·kg^−1^, mass: 76.2 ± 14.1 kg, height: 177 ± 11 cm, weekly training volume: 10.3 ±  ± 3.4 h·week^−1^). A priori sample size estimation indicated that only four participants per group were required to detect a statistically significant change in power output at VT_1_ after 2 h of exercise at 90% of VT_1_ measured at rested state using data from similar endurance‐trained cohort with 80% statistical power (Stevenson et al. 2022). Inclusion criteria were: habitually training > 7 h week^−1^, free of viral infection and musculoskeletal injury for 3 months, not suffering from cardiovascular disease, and able to self-report a record power output over 20 min of > 3.5 W kg^−1^ body mass for males or > 2.5 W kg^−1^ body mass for females. These criteria were formally assessed through a health screening questionnaire during the first laboratory visit. The Auckland University of Technology Ethics Committee approved all procedures (23/37), and all participants provided written informed consent.

### Study design

**Fig. 1:**
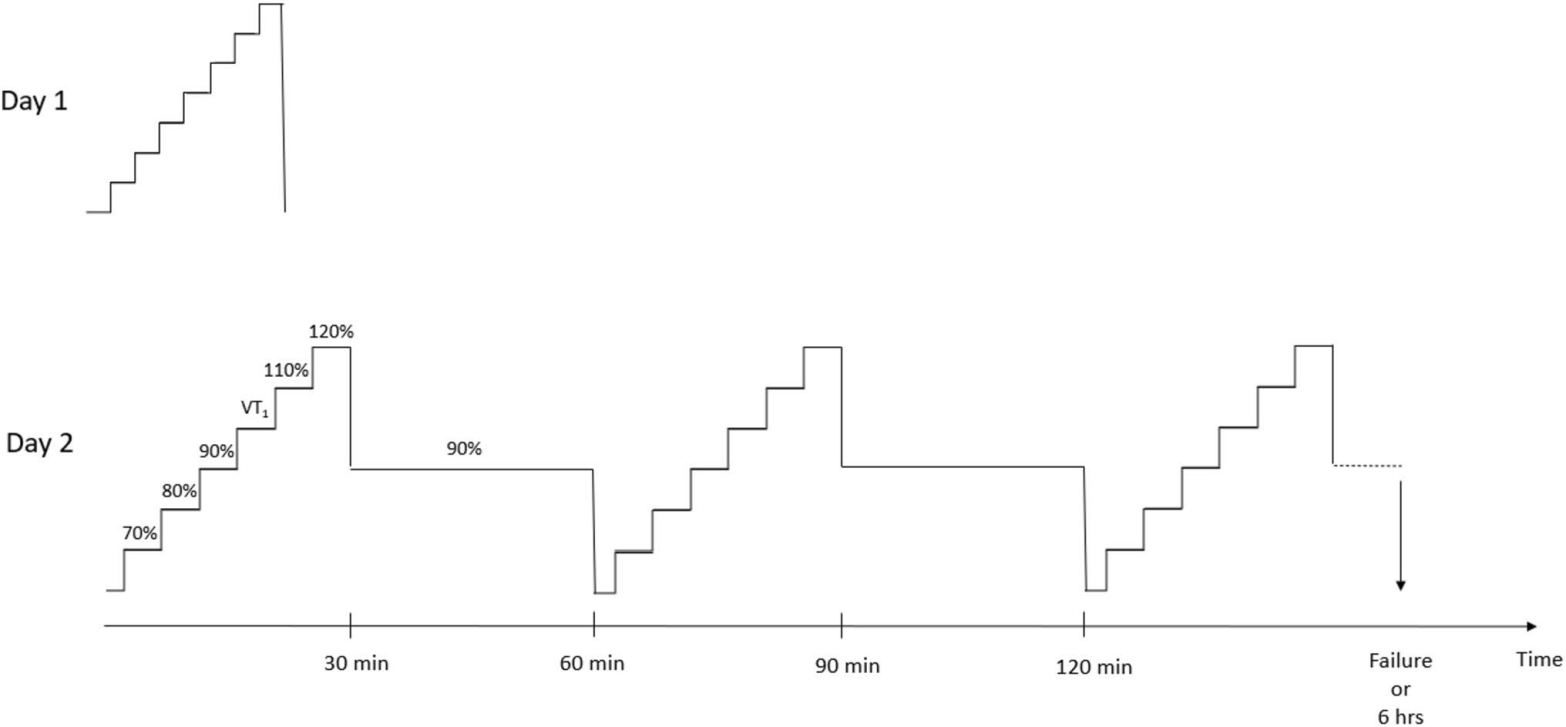
Schematic representation of study design. On day 2, the incremental exercise test and the 30 min bout at 90% of estimated power output at VT_1_ were repeated until failure, or for a maximum of 6 h. Percentage values represent the intensity of each phase referring to the power output at VT_1_ estimated in the first visit

An overview of the study design is shown in Fig. 1. Participants visited the laboratory on two occasions. The first visit was to determine the peak rate of oxygen uptake ($\dot{\text{V}}$O_2_peak), the peak fat oxidation rate (PFO), and to estimate the power output at first (VT_1_) and second (VT_2_) ventilatory thresholds. The estimated VT_1_ was used to set the intensities in the second visit. In line with previous studies, VT_1_ was used as marker of the moderate-to-heavy intensity transition. (Stevenson et al. 2022) In the second visit, VT_1_ was assessed in the rested state and then again each hour until exhaustion (up to a maximum of 6 h) to observe the time course of its decrease during prolonged exercise, the association between the time to a 5% decrease in VT_1_ (Δ5%VT_1_) and time-to-task failure (TTTF), and the physiological correlates of Δ5%VT_1_ and TTTF. Laboratory tests were performed on participants’ own road bicycle mounted on a direct-drive indoor cycling trainer (Kickr v5, Wahoo Fitness, Atlanta, USA), which provides valid and reliable measurements of power output (Hoon et al. 2016).

### Visit one: characterisation trial

Participants arrived in the laboratory having fasted overnight for ~ 10 h, ingested 1.5 L of water in the morning before arrival, and refrained from caffeine, vigorous exercise, and alcohol for 24 h. Height and body mass was recorded. Participants then completed a maximal incremental test. For males, cycling commenced at 95 W, with the power output increasing by 35 W every 3 min. Females started at 75 W and power output was increased by 25 W every 3 min. The discrepancy in step increments between females and males was chosen to ensure a dense cluster of datapoints around VT_1_ and VT_2_, given that lower absolute power at VT_1_ in females compared to males is usually recorded (Kim et al. 2021). Expired gases were collected continuously using indirect calorimetry with mixing chamber technology (TrueOne 2400, ParvoMedics, UT, USA). When the respiratory exchange ratio (RER) reached 1.0, the power output was increased by 35 W (males) or 25 W (females) every minute until volitional exhaustion. The $\dot{\text{V}}$O_2_peak was accepted as the highest 15-s average $\dot{\text{V}}$O_2_. The estimated VT_1_ and VT_2_ was identified as the $\dot{\text{V}}$O_2_ at which a systematic rise in $\dot{\text{V}}$_E_ ˙$\dot{\text{V}}$O_2_^−1^ and $\dot{\text{V}}$_E_ $\dot{\text{V}}$CO_2_^−1^ occurred, respectively. These $\dot{\text{V}}$O_2_ values were converted to power output by linear fit of the power output vs. $\dot{\text{V}}$O_2_ relationship, using the last minute of $\dot{\text{V}}$O_2_ data from each 3-min stage (Stevenson et al. 2022). The $\dot{\text{V}}$O_2_ at VT_1_ and VT_2_ values were picked independently by two operators with multiple years’ experience, and the average between the two values was considered (mean difference: 1 ± 6 W, range: 0–20 W). Assessment of the first ventilatory threshold using ventilatory equivalents has excellent reliability (3.5%) (Pallares et al. 2016). The last minute of expired gas data in each 3-min stage was also used to quantify whole-body fat oxidation rates and gross efficiency (GE) using standard stoichiometric equations (Jeukendrup and Wallis 2005; Hopker et al. 2009) (Eq. 1). The highest observed rate of whole-body fat oxidation was identified as PFO. The stage closest to the estimated VT_1_ was used to characterise GE.

### Visit two: prolonged exercise assessment

Participants arrived in the laboratory ~ 3–7 days later, having consumed a breakfast containing ~ 2 g kg^−1^ of carbohydrate (in line with general guidelines about pre-exercise meal, Podlogar and Wallis 2022) and ~ 800 mL of water 1 h beforehand, and having refrained from caffeine, vigorous exercise, and alcohol for 24 h. Cycling began with a 3-min warm-up at 100 W. Subsequently, VT_1_ was measured using a 6 × 4 min incremental test, with continuous collection of expired gases. The 4 min steps were used to increase the density of datapoints are VT_1_. The intensities of the six increments were: 70%, 80%, 90%, 100%, 110%, and 120% of the VT_1_ power output estimated in the first laboratory visit. The VT_1_ was determined using the same methods outlined in the first laboratory visit, but with greater precision given the denser cluster of datapoints around the transition. This method has been used to estimate the moderate-to-heavy intensity transition previously, producing similar results to blood lactate-derived measurements (Stevenson et al. 2022). After the initial incremental test, participants undertook repetitions of 30 min at 90% of the power output associated with VT_1_ in the first visit, and the 30-min incremental test, to calculate power output at VT_1_ each hour until exhaustion. Maximum duration was set at 6 h even if failure was not attained. Task failure was defined volitionally or through inability to maintain a pedalling cadence ≥ 50 rpm despite encouragement. ‘Time 0’ for calculating time-to-task failure was the beginning of the warm-up before the first incremental step test. Expired gases were collected throughout all incremental tests, and the $\dot{\text{V}}$O_2_ and $\dot{\text{V}}$CO_2_ in the last minute of each of the six 4-min stages was used to calculate the respiratory exchange ratio (RER), and rates of fat and carbohydrate oxidation using stoichiometric equations (Eq. 1) (Jeukendrup and Wallis 2005). During each 30 min bout at 90% of estimated VT_1_, participants consumed 600 mL of water in a solution made with an electrolyte mix (Science in Sport GO Hydro). Participants were instructed to adopt their preferred cadence throughout the trial.

Energy expenditure (kcal⋅min^−1^) = (0.55⋅$\dot{\text{V}}$CO_2_) + (4.471⋅$\dot{\text{V}}$O_2_).

GE (%) = Work performed (kcal⋅min^−1^) / energy expenditure (kcal⋅min^−1^) ⋅ 100.

Fat oxidation (g⋅min^−1^) = 1.695⋅$\dot{\text{V}}$O_2_ -1.701⋅$\dot{\text{V}}$CO_2_.

CHO oxidation (g⋅min^−1^) = 4.21⋅$\dot{\text{V}}$CO_2_ -2.962⋅$\dot{\text{V}}$O_2_.

Equation 1 where $\dot{\text{V}}$O_2_ and $\dot{\text{V}}$CO_2_ are in L⋅min^−1^.

To quantify the contribution of changes in metabolic energy expenditure (EE) and gross efficiency to the decrease in VT_1_ power output, for each participant the power output that would be attained using the rate of metabolic energy expenditure observed at VT_1_ in the last step test (i.e., before failure or the 6 h timepoint) and the GE at VT_1_ measured in the first step test (rested state) was calculated (lastEErestGE) (Stevenson et al. 2022). For each step test, metabolic EE and GE at VT_1_ were calculated using linear regression of power output vs. the EE or GE in the last minute of each step. Then, the proportion of prolonged exercise-induced changes in VT_1_ power output associated with changes in energetic efficiency and rates of metabolic energy expenditure achieved at the transition was calculated using the below equations (Eq. 2).

Contribution of change in energetic efficiency to change in power output at VT_1_ = (laststep – lastEErestGE)*100/ΔlastrestVT_1_.

Contribution of change in energetic efficiency to change in power output at VT_1_ = (lastEErestGE–restedstate)*100/ΔlastrestVT_1_.

Equation 2 where laststep = power output at VT_1_ in the last step test before task failure or reaching the 6 h timepoint; restedstate = power output at VT_1_ measured in the first step test; lastEErestGE = power output at VT_1_ that would be produced in the last step test assuming the gross efficiency observed at VT_1_ in the first step test; ΔrestlastVT_1_ = the difference between first and last step VT_1_ power output.

The metabolic cart was calibrated against known gas volumes and concentrations prior to the start of the trial. However, the metabolic cart was not recalibrated during the trial. While it is possible some calibration drift occurred, we do not anticipate that this meaningfully altered our primary outcome measures, which was VT_1_ as determined by breakpoints in the relationship between ventilatory equivalents and oxygen consumption during individual incremental tests, and initial substrate oxidation rates.

### Statistical analysis

Data are expressed as mean ± standard deviation, unless otherwise stated. Statistical significance was inferred when *p* ≤ 0.05. The normality of datasets was assessed using the Shapiro–Wilk test. The effect of time on power output at VT_1_ was assessed with a repeated measures one-way analysis of variance, using the three timepoints recorded for all participants (rested state, 1 h, and the measurement preceding failure). Variance was located post-hoc using paired *t* tests. For each individual, the progression of power output at VT_1_ against time was fitted to linear, exponential, and second-order polynomial functions. For each VT_1_ measurement, the timepoint allocated was the time at the beginning of the incremental test. The curve with best model fit using the method of least squares (R^2^) was selected. These curves were used to estimate the time to a 5% reduction in power output at VT_1_ vs. the initial value (Δ5%VT_1_). The normality of these datasets was assessed. The relationship between Δ5%VT_1_ and TTTF was assessed using a Spearman’s rank-order (r_s_) correlation, and expressed with 95% confidence intervals. Bivariate relationships were also assessed between these variables (Δ5%VT_1_ and TTTF) and a selection of physiological variables measured in the first ($\dot{\text{V}}$O_2_peak, VT_1_, VT_2_, GE at VT_1_, the percentage of $\dot{\text{V}}$O_2_peak at which VT_1_ and VT_2_ occurred, PFO, RER and substrate oxidation rates at VT_1_) and second (RER and substrate oxidation rates during the first four stages of the initial incremental test) visits using Pearson’s product-moment correlations (r) or Spearman’s rank order correlations (r_s_), depending on normality. Post-hoc Bonferroni correction was used for the correlations including variables related to the first four stages of the initial incremental test of day 2 to reduce the risk inflating the overall chance of a type I error (Curtin and Schultz 1998). The magnitude of correlations was assessed using the following benchmarks: < 0.10, trivial; 0.10–0.29, small; 0.30–0.49, moderate; > 0.50, large (Cohen 1992). All data were analysed using SPSS version 29.0.1.0.

## Results

### Baseline physiological characteristics

Physiological characteristics measured during the first characterisation trial were: $\dot{\text{V}}$O_2_peak, 52.3 ± 5.2 mL·min^−1^·kg^−1^; VT_1_, 189 ± 35 W; VT_2_ 256 ± 48 W; GE at VT_1_ 23.3 ± 1.7%; VT_1_ (% of $\dot{\text{V}}$O_2_peak), 64 ± 3%; VT_2_ (% of $\dot{\text{V}}$O_2_peak), 84 ± 4%; PFO, 0.58 ± 0.11 g⋅min^−1^.

### Time course of changes in the power at the moderate-to-heavy transition

**Fig. 2:**
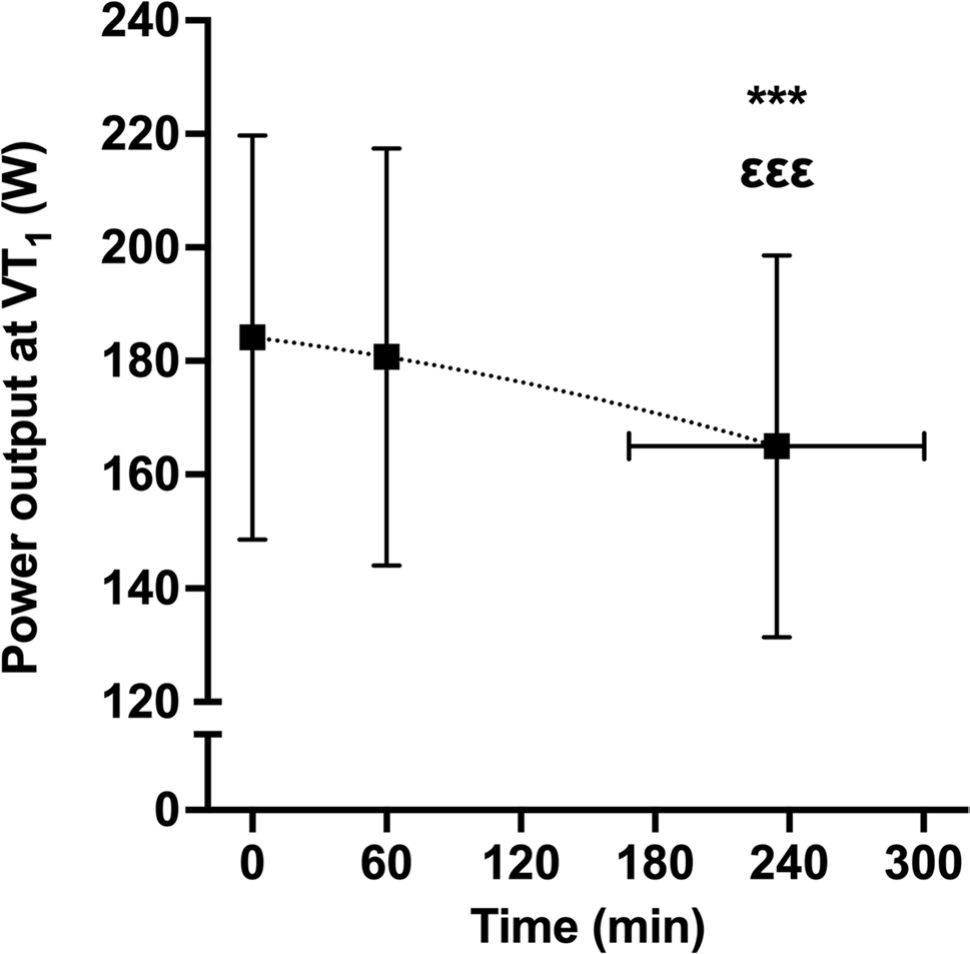
Group-level relationship between power output at the first ventilatory threshold (VT1) and time. ***denotes *p* < 0.001 vs. rested state, εεε denotes *p* < 0.001 vs. after 1 h

**Fig. 3:**
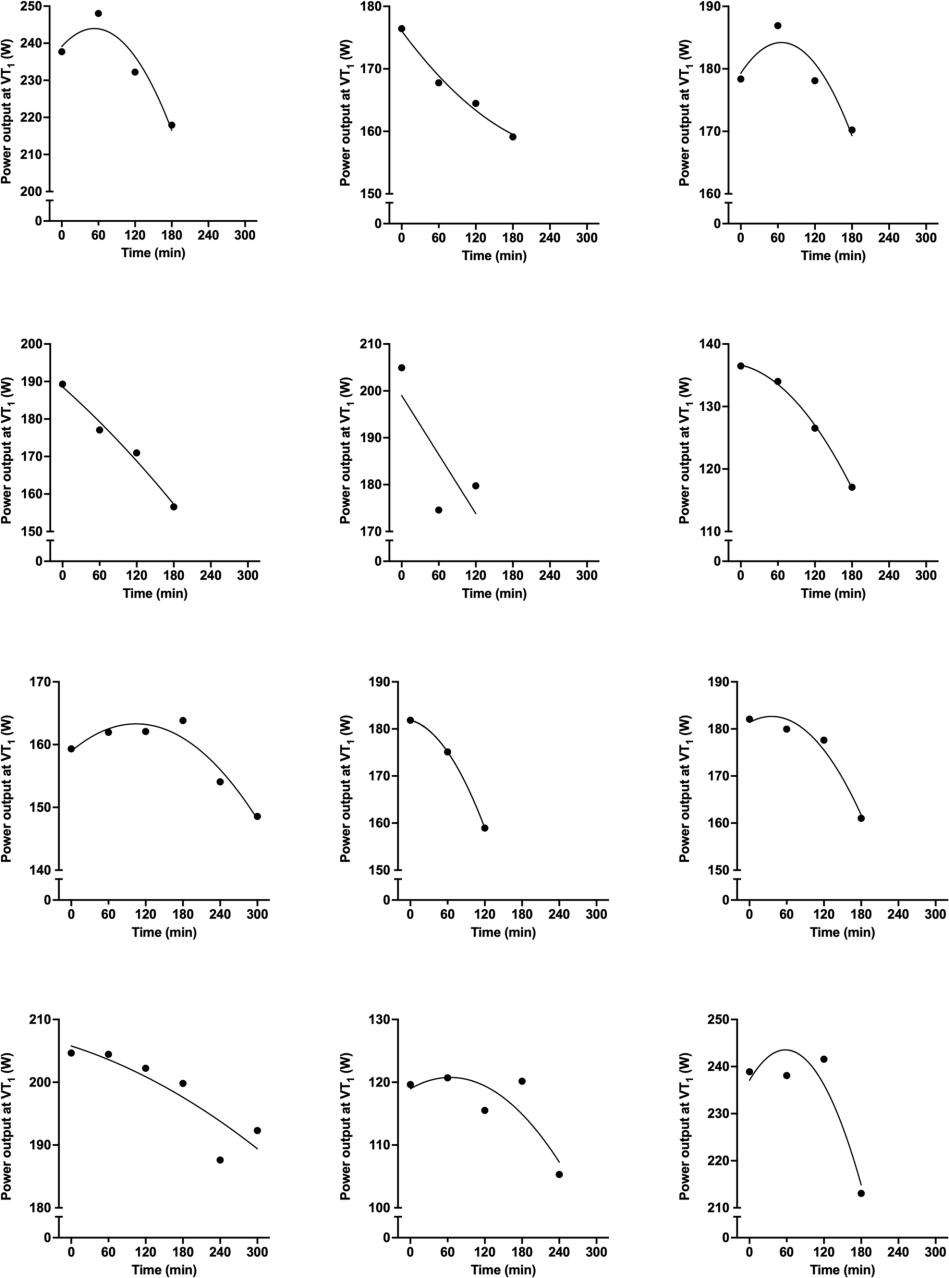
Individual-level relationships between power output at the first ventilatory threshold (VT1). and time. ***denotes *p* < 0.001 vs. rested state, εεε denotes  < 0.001 vs. after 1 h

Power output at VT_1_ measured in the first visit (189 ± 35 W) and in the rested state in the second visit (184 ± 35 W) were similar (*p* = 0.213) and significantly related (*r* = 0.948, 95% CI, 0.821–0.986, large, *p* < 0.001). There was a significant effect of time (*p* < 0.001) on power output at VT_1_ measured during the second visit. Power output at VT_1_ was unchanged after an hour (rested state, 184 ± 35 W vs. 1 h, 181 ± 37 W, *p* = 0.290), but significantly decreased in the test prior to task failure (165 ± 34 W, *p* < 0.001 vs. rested state and 1 h, Fig. 2). The relationship between power output at VT_1_ and time was best fit to a second-order polynomial function in 11 out of 12 participants, with the remaining participant best fit to a linear function. The curve-fitting of second-order polynomial functions to individual-level relationships between power output at VT_1_ and time was strong (*N* = 12, *R*^2^ = 0.92 ± 0.09, range: 0.72–1.00, Fig. 3). Both energy expenditure (*p* = 0.001) and gross efficiency (*p* = 0.02) at VT_1_ were reduced in the test prior to task failure compared to the rested state (− 7.3 ± 6.2%, range: − 0.4 to − 19.3% and − 4.1 ± 5.2%, range: − 10.6 to + 5.0%). The contribution of change in energy expenditure and energetic efficiency to change in power output at VT_1_ between the test prior to task failure and at rested state was 64/36 ± 43% When considering the steps at 90% of VT_1,_ pedalling cadence did not significantly change (*p* = 0.528) between rested state (82 ± 9 rpm), after an hour (80 ± 9 rpm) and in the test prior to task failure (78 ± 9 rpm).

### Relationship between durability of the moderate-to-heavy intensity transition and time-to-task failure

**Fig. 4:**
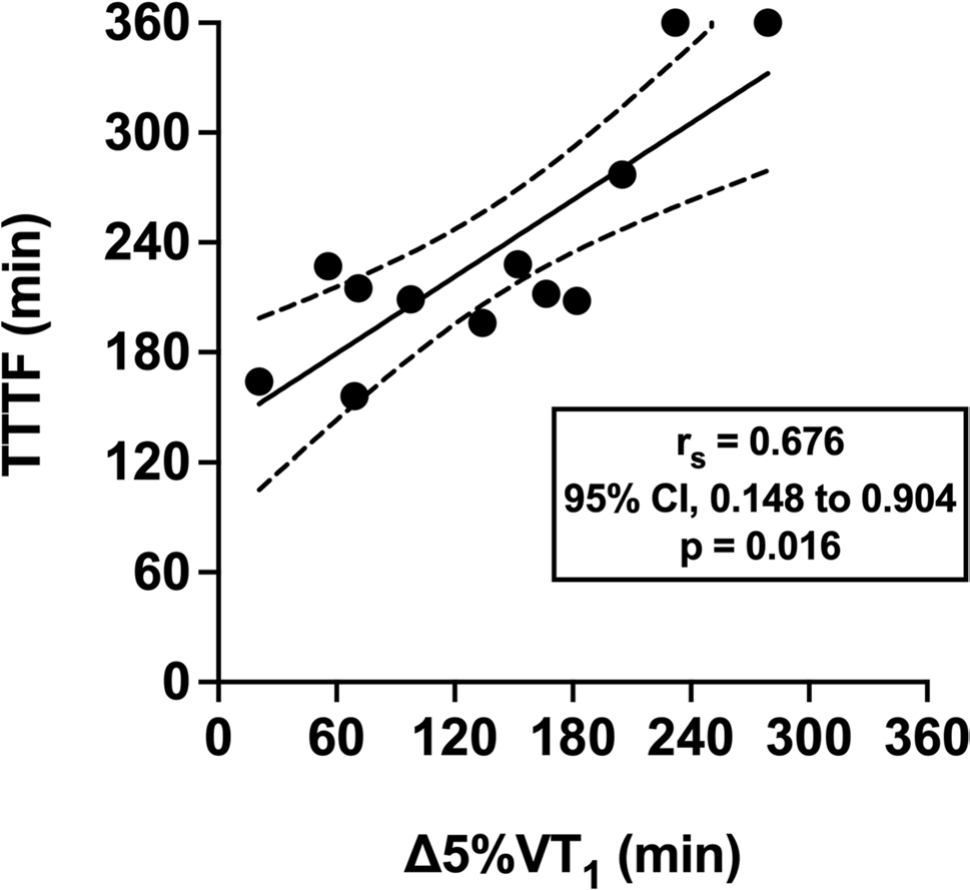
Relationship between time-to-task failure (TTTF) and estimated time to a 5% reduction in power output at the moderate-to-heavy intensity transition (∆5%VT_1_)

The TTTF was 234 ± 66 min (range: 164–360 min). The time to Δ5%VT_1_ was 139 ± 78 min (range: 21–279 min). The TTTF was significantly correlated with Δ5%VT_1_ (*r*_s_ = 0.676, 95% CI, 0.148–0.904, large, *p* = 0.016, Fig. 4).

### Physiological correlates of durability of the moderate-to-heavy intensity transition and time-to-task failure

**Fig. 5:**
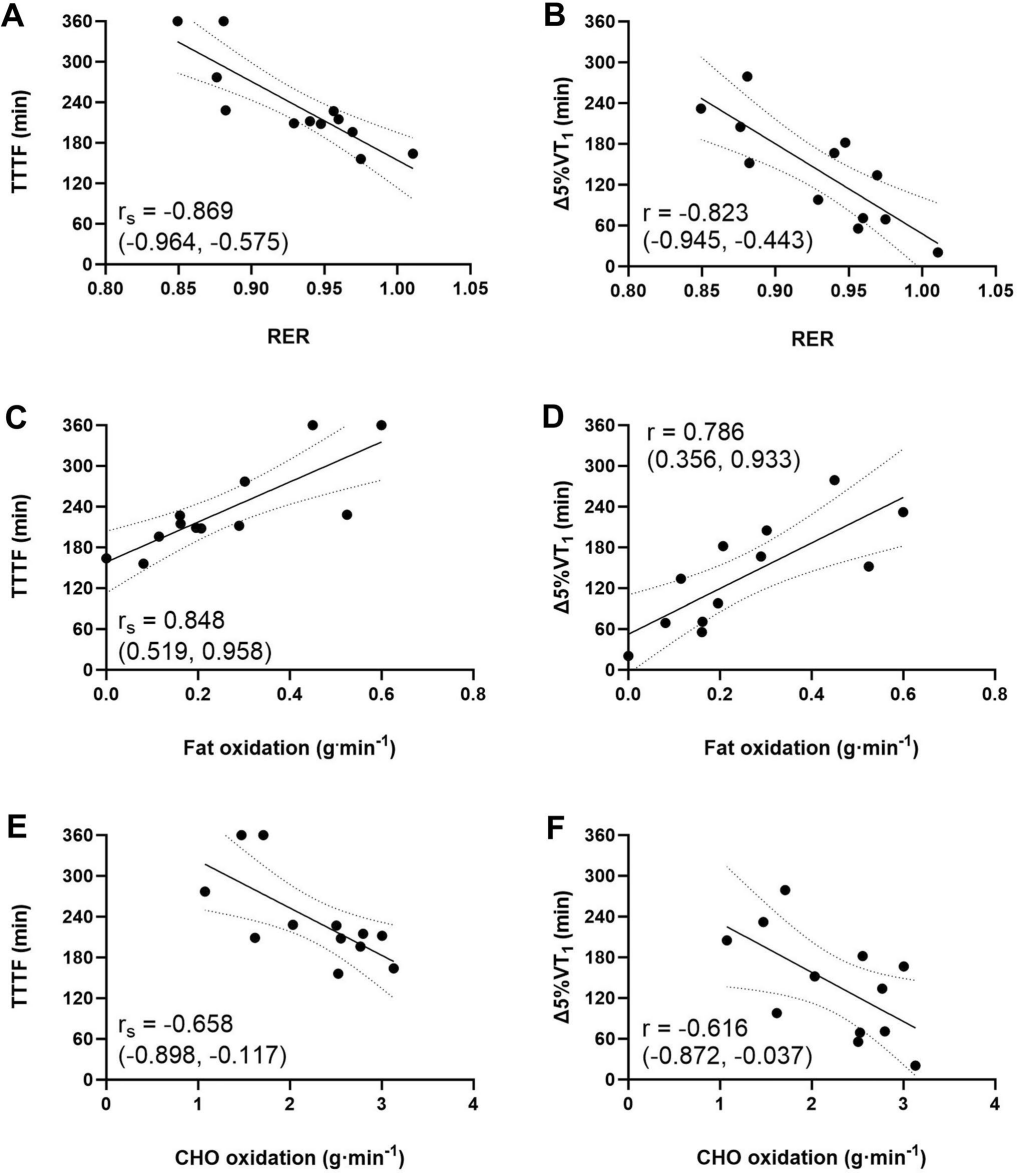
Bivariate relationships between time-to-task failure (TTTF) or estimated time to a 5% reduction in power output at the moderate-to-heavy intensity transition (∆5%VT_1_) and respiratory exchange ratio (RER) (**A**, **B**), absolute fat oxidation rates (**C**, **D**), and absolute carbohydrate (CHO) oxidation rates (**E**, **F**) measured during the initial incremental exercise test of the experimental trial (visit two). Data refers to the third step of the initial incremental exercise test, which corresponded to 90% of the power output at the first ventilatory threshold (VT_1_) estimated in the first laboratory visit. Pearson (r) and Spearman’s rank-order (r_s_) correlation coefficients with 95% confidence intervals in brackets are reported

The TTTF and Δ5%VT_1_ were not significantly correlated with the traditional physiological parameters measured in the first visit ($\dot{\text{V}}$O_2_peak, power output at VT_1_ or VT_2_, GE at VT_1_, or the percentage of $\dot{\text{V}}$O_2_peak at which VT_1_ or VT_2_ occurred) or to PFO and substrate oxidation variables in the step below VT_1_ measured in the first visit (Table 1). Significant large correlations were found between TTTF and Δ5%VT_1_ and relative and absolute fat oxidation rates measured during specific 4-min stages of the first incremental test in the second visit (Table 2, Fig. 5).

**Table 1:** Bivariate relationships various baseline physiological parameters measured during the first characterisation trial and time-to-task failure (TTTF) and estimated time to a 5% reduction in power output at the moderate-to-heavy intensity transition (∆5%VT_1_)

	TTTF	∆5%VT_1_
$\dot{\text{V}}$O_2_peak (mL.kg^−1.^min^−1^)	*r*_s_ = 0.168 (− 0.464, 0.687) *p* = 0.601	*r* = − 0.287 (− 0.739, 0.343) *p* = 0.366
VT_1_ (W)	*r*_s_ = 0.104 (− 0.514, 0.651 *p* = 0.748	*r* = − 0.050 (− 0.607, 0.539) *p* = 0.877
VT_2_ (W)	*r*_s_ = 0.181 (− 0.454, 0.694) *p* = 0.574	*r* = 0.045 (− 0.543, 0.604) *p* = 0.889
GE at VT_1_ (%)	*r*_s_ = 0.228 (− 0.414, 0.718) *p* = 0.477	*r* = 0.192 (− 0.429, 0.690) *p* = 0.55
VT_1_ (%$\dot{\text{V}}$O_2_peak)	*r*_s_ = − 0.309 (-0.758, 0.339) *p* = 0.328	*r* = − 0.151 (− 0.667, 0.463) *p* = 0.638
VT_2_ (%$\dot{\text{V}}$O_2_peak)	*r*_s_ = − 0.200 (− 0.704–0.438) *p* = 0.534	*r*_s_ = 0.068 (− 0.540, 0.630) *p* = 0.833
PFO (g⋅min^−1^)	*r*_s_ = 0.340 (− 0.308, 0.773) *p* = 0.279	*r* = 0.241 (− 0.386, 0.716) *p* = 0.450
RER at VT_1_	*r*_s_ = − 0.501 (− 0.889, 0.066) *p* = 0.140	*r*_s_ = − 0.555 (− 0.878, 0.115) *p* = 0.096
Fat oxidation at VT_1_ (g⋅min^−1^)	*r*_s_ = 0.455 (− 0.245, 0.843) *p* = 0.191	*r*_s_ = 0.509 (− 0.178, 0.862) *p* = 0.133
CHO oxidation at VT_1_ (g⋅min^−1^)	*r*_s_ = − 0.296 (− 0.761, 0.369) *p* = 0.377	*r*_s_ = − 0.397 (− 0.771, 0.348) *p* = 0.227

*Δ5%VT1* estimated time to a 5% reduction in power output at the first ventilatory threshold, *CHO* carbohydrates, *GE* gross efficiency, *RER* respiratory exchange ration, *TTTF* time-to-task failure, $\dot{\text{V}}$*O*_*2*_*peak* peak oxygen consumption, *VT*_*1*_ first ventilatory threshold, *VT*_*2*_ second ventilatory threshold

**Table 2:** Bivariate relationships between substrate utilisation parameters measured during the initial step test of visit 2 and time-to-task failure (TTTF) and durability of the power at the moderate–heavy transition, expressed as estimated time to a 5% reduction in power output at the moderate-to-heavy intensity transition (Δ5%VT_1_)

	TTTF (min)	Δ5%VT_1_ (min)
RER at 70% VT_1_	*r*_s_ = − 0.756* (− 0.936, − 0.267) *p* = 0.020	*r* = − 0.698 (− 0.0.134) *p* = 0.068
RER at 80% VT_1_	*r*_s_ =− 0.907*** (− 0.975, − 0.685) *p* < 0.001	*r* = − 0.687 (− 0.899, − 0.157) *p* = 0.056
RER at 90% VT_1_	*r*_s_ = − 0.869*** (− 0.964, − 0.575) *p* < 0.001	*r* = − 0.823** (− 0.940.443) *p* = 0.004
RER at VT_1_	*r*_s_ = − 0.830** (− 0.953, − 0.475) *p* = 0.008	*r* = − 0.859***(− 0.956, − 0.534) *p* < 0.001
Fat oxidation at 70% VT_1_ (g⋅min^−1^)	*r*_s_ = 0.788* (0.339, 0.945) *p* = 0.016	*r* = 0.591 (− 0.043, 0.872) *p* = 0.220
Fat oxidation at 80% VT_1_ (g⋅min^−1^)	*r*_s_ = 0.837*** (0.492,0.955) *p* < 0.001	*r* = 0.617 (0.039,0.873) *p* = 0.128
Fat oxidation at 90% VT_1_ (g⋅min^−1^)	*r*_s_ = 0.848*** (0.519,0.958) *p* < 0.001	*r* = 0.786** (0.356, 0.933) *p* = 0.008
Fat oxidation at VT_1_ (g⋅min^−1^)	*r*_s_ = 0.837*** (0.492,0.955) *p* < 0.001	*r* = 0.783* (0.350,0.932) *p* = 0.012
CHO oxidation at 70% VT_1_ (g⋅min^−1^)	*r*_s_ = − 0.656 (− 0.905, − 0.072) *p* = 0.114	*r* = − 0.551 (− 0.858, 0.101) *p* = 0.0316
CHO oxidation at 80% VT_1_ (g⋅min^1^)	*r*_s_ = − 0.662 (− 0.899, − 0.123) *p* = 0.076	*r* = − 0.523 (− 0.839, 0.089) *p* = 0.308
CHO oxidation at 90% VT_1_ (g⋅min^1^)	*r*_s_ =− 0.658 (− 0.898, − 0.117) *p* = 0.080	*r* = − 0.616 (− 0.872, − 0.037) *p* = 0.132
CHO oxidation at VT_1_ (g⋅min^1^)	*r*_s_ = − 0.543 (− 0.857, 0.064) *p* = 0.272	*r* = − 0.592 (− 0.863, 0.000) *p* = 0.172

Data are reported as Pearson’s product-moment (r) or Spearman’s rank-order (r_s_) correlation coefficients (95% confidence intervals), with accompanying P values

*Δ5%VT*_*1*_ estimated time to a 5% reduction in power output at the first ventilatory threshold, *CHO* carbohydrate, *RER* respiratory exchange ratio, *TTTF* time-to-task failure, *VT*_*1*_ first ventilatory threshold

^*^p < 0.05, **p < 0.01, ***p < 0.001

## Discussion

The primary observations of this study were: (i) the time course of the decrease in power output at the moderate-to-heavy transition during prolonged exercise was non-linear and best fit to a second-order polynomial function, with the time of onset differing substantially at an inter-individual level; (ii) the time of onset of the decrease in power output at the moderate-to-heavy intensity transition during prolonged exercise was positively correlated with exercise capacity; (iii) durability of the moderate-to-heavy intensity transition and exercise capacity were positively related to relative (measured through the respiratory exchange ratio) and absolute rates of fat oxidation in the initial periods of prolonged exercise. These data, therefore, provide the first support for the hypothesis that durability of the moderate-to-heavy intensity transition is an important determinant of exercise capacity in the moderate–heavy intensity domain, and support the hypothesis that durability is related to fat oxidation rates in the initial stages of prolonged exercise.

### Time course of decrease in power at the moderate–heavy transition during prolonged exercise

The power output at the moderate-to-heavy intensity transition decreased in a non-linear fashion during prolonged exercised (Fig. 1a and b). Specifically, power output at the moderate-to-heavy intensity transition was unchanged compared to baseline values after an hour of exercise, but was decreased significantly in the assessment immediately prior to task failure (Fig. 2a). The relationship between power output at the moderate-to-heavy intensity transition and time was best fit to a second-order polynomial function in 11 of 12 participants, and the fit of individual curves was strong (*R*^2^ = 0.92 ± 0.09, range: 0.72–1.00, Fig. 2b). These data suggest a ‘threshold’ after which power output at the moderate-to-heavy intensity transition decreases, and our data demonstrate substantial inter-individual variability in the time of onset of this decrease (Δ5%VT_1_, range: 21–279 min). These data advance previous work, which simply reported a reduction of variable magnitude in power output at the moderate-to-heavy intensity transition following 2.5 h of cycling (Stevenson et al. 2022). Therefore, these data support calls for identification of a physiological marker that can be measured in real-time and used to assess proximity to the moderate-to-heavy intensity transition during exercise for within-session intensity regulation, training load monitoring, and training programming. The decrease in power output at VT_1_ between the first step test and the test prior to task failure was due to decreased metabolic energy expenditure and gross efficiency, in line with a previous study (Stevenson et al. 2022).

### Durability of physiological parameters and exercise capacity

A further novel observation in the present investigation was that of a strong positive relationship between durability of the moderate-to-heavy intensity transition (Δ5%VT_1_) and exercise capacity (Fig. 3). Therefore, these data are the first to support the hypothesis that durability of the moderate-to-heavy intensity transition is an important determinant of outcomes relevant to competition in prolonged endurance events. Whilst the relevance of exercise capacity tests to real world performance has been questioned, we contend that the ability to maintain a certain power output can be considered relevant for success in different endurance competitions, such as road cycling, long distance triathlon and ultra-cycling. Exercise capacity may also be an important component of training in programmes designed to stimulate adaptation through high training volumes (Laursen 2010). Regardless, we recommend that future studies investigate relationships between durability of the moderate-to-heavy intensity transition and other endurance performance measures, such as the effect of prolonged exercise on severe-intensity performance. These ‘performance durability’ measures have been related to competition outcomes in under 23 and professional road cycling (Van Erp et al. 2021; Gallo et al. 2022; Muriel et al. 2022).

Mechanistically, the association between durability of the moderate-to-heavy intensity transition and exercise capacity may be attributable to effects on time spent in the heavy-intensity domain during prolonged exercise. Less durable athletes with larger and more rapid reductions in power output at the moderate-to-heavy intensity transition would have more rapidly accumulated time in the heavy-intensity domain. This is despite the experimental protocol having been fixed according to the physiological parameters estimated in the characterisation trial. Heavy-intensity exercise results in greater extracellular K^+^ accumulation than moderate-intensity exercise (Black et al. 2017). Extracellular K^+^ accumulation has been shown to depress muscle force production and, therefore, induce fatigue in vitro (Cairns et al. 1997; de Paoli et al. 2007). Therefore, it is possible the less durable athletes fatigued faster due to earlier heavy-intensity exercise-induced extracellular K^+^ accumulation. As we did not measure extracellular [K^+^], we recommend that future studies explore this mechanism directly.

### Physiological correlates of durability

We did not observe relationships between traditional physiological profiling parameters measured in a rested state and durability of the moderate-to-intensity transition. This aligns with previous studies assessing relationships between these parameters and durability of the moderate-to-intensity transition (Stevenson et al. 2022) or durability of maximal mean power outputs in the severe intensity domain (Passfield et al. 2000; Valenzuela et al. 2022). These data, and the inter-individual variability in the present study, therefore, suggest that durability during prolonged moderate-to-heavy intensity exercise is an independent physiological parameter. Only one previous study has reported a positive correlation between traditional physiological markers and a durability metric, in that case durability of the heavy-to-severe intensity transition (Spragg et al. 2023). This discrepancy could be related to the differences in the intensity of the prolonged fatiguing exercise, which could underlie different physiological mechanism determining durability.

A further novel finding of the present study was that durability of the moderate-to-heavy intensity transition was strongly and positively related to absolute and relative (measured through RER) rates of fat oxidation during the initial incremental test of the experimental trial (Table 2, Fig. 5) This aligns with our hypothesis, and previous work reporting relationships between prolonged exercise-induced glycogen depletion and durability of the heavy-to-severe intensity transition (Clark et al. 2019). Mechanistically, this may be attributable to better preservation of glycogen in athletes oxidising fat at higher rates. Muscle glycogen depletion reduces the number of excitable fibres (Cairns and Renaud 2023). It is, therefore, possible that athletes oxidising fat at higher rates more effectively maintained muscle glycogen availability, and, therefore, completed more exercise prior to impaired function of individual, glycogen-depleted fibres. This aligns with the decreased metabolic energy expenditure and gross efficiency at VT_1_ observed between the test prior to task failure and the rested state. The decreased metabolic energy expenditure at VT_1_ could be due to progressive inexcitability of type I muscle fibres, while the decreased gross efficiency may have occurred due to compensatory recruitment of less efficient type II muscle fibres (Jones et al. 2011). This mechanism aligns with the non-linear nature of the reduction in power output at the moderate-to-heavy intensity transition, and the absence of an effect of 1 h of exercise on power output at the moderate-to-heavy intensity transition (Fig. 2a and b). Future studies should assess relationships between glycogen availability, specifically intramyofibrillar glycogen availability, and durability of the moderate-to-heavy intensity transition to examine this hypothesis directly.

As in previous work (Stevenson et al. 2022), durability of the moderate-to-heavy intensity transition was not related to PFO. As PFO occurred at a lower power output (65 ± 14% of estimated VT_1_) than the range of intensities performed during experimental trial (70–120% of estimated VT_1_), our data suggests durability is related to the capacity to maintain high fat oxidation rates at the specific intensities of the prolonged exercise protocol.

Even if moderate-to-large correlation coefficients between durability and relative and absolute fat oxidation rates at VT1 measured during day 1 were observed, these were not significant (Table 1). This could be related to interindividual differences in the variability of substrate utilization between days 1 and 2, that could be due to differences in macronutrients intake between the two lab visits (Burke LM 2), or even to different suppression of fat oxidation following standardized carbs ingestion pre-exercise (day 2) compared to fasting (day 1).

Despite the putative mechanism, these data suggest caution should be taken when making considerations about durability, exercise capacity and substrates utilization based on tests performed in different days and without standardizing chronic and acute nutritional status.

## Conclusions

In summary, we present here novel data that: (i) elucidates the non-linear time course of the reduction in power output at the moderate-to-heavy intensity transition during prolonged exercise, (ii) identifies a relationship between durability of the moderate-to-heavy intensity transition and exercise capacity, and (iii) supports the hypothesis that durability of the moderate-to-heavy intensity transition is related to initial absolute and relative rates of fat oxidation at the specific intensities of subsequent prolonged exercise. We contend that these data collectively support calls for identification of a physiological marker that can be measured in real-time and used to assess proximity to the moderate-to-heavy intensity transition during exercise for within-session intensity regulation, training load monitoring, and training programming. We recommend that future research explores the mechanisms that underpin durability of the moderate-to-heavy intensity transition. Specifically, we recommend mechanisms related to extracellular [K^+^] accumulation and intramyofibrillar glycogen depletion are investigated directly. We also recommend that relationships between durability of the moderate-to-heavy intensity transition and endurance performance outcomes, such as ‘performance durability’ in the severe-intensity domain and prolonged time trial performance are explored. Finally, future studies could investigate the effects of adaptations to fatty acid metabolism on durability, and the effectiveness of different interventions for enhancing durability.

## Data Availability

Data is available from the corresponding author upon reasonable request.

## Abbreviations

CHO: Carbohydrates
GE: Gross efficiency
PFO: Peak fat oxidation
RER: Respiratory exchange ratio
TTTF: Time to task failure
$\dot{\text{V}}$CO_2_: Rate of carbon dioxide production
$\dot{\text{V}}$E: Rate of ventilation
$\dot{\text{V}}$O_2_: Rate of oxygen production
$\dot{\text{V}}$O_2_max: Maximal oxygen uptake
$\dot{\text{V}}$O_2_peak: Peak rate of oxygen consumption
VT_1_: First ventilatory threshold
VT_2_: Second ventilatory threshold
